# Highly Pathogenic Reassortant Avian Influenza A(H5N1) Virus Clade 2.3.2.1a in Poultry, Bhutan

**DOI:** 10.3201/eid2212.160611

**Published:** 2016-12

**Authors:** Atanaska Marinova-Petkova, John Franks, Sangay Tenzin, Narapati Dahal, Kinzang Dukpa, Jambay Dorjee, Mohammed M. Feeroz, Jerold E. Rehg, Subrata Barman, Scott Krauss, Pamela McKenzie, Richard J. Webby, Robert G. Webster

**Affiliations:** St. Jude Children’s Research Hospital, Memphis, Tennessee, USA (A. Marinova-Petkova, J. Franks, J.E. Rehg, S. Barman, S. Krauss, P. McKenzie, R.J. Webby, R.G. Webster);; National Centre for Animal Health, Ministry of Agriculture and Forests, Thimphu, Bhutan (S. Tenzin, N. Dahal, K. Dukpa, J. Dorjee);; Jahangirnagar University, Dhaka, Bangladesh (M.M. Feeroz)

**Keywords:** highly pathogenic avian influenza H5N1, clade 2.3.2.1a, Bhutan, Indian subcontinent, reassortant, outbreak, transmission, ferret model: respiratory diseases, influenza virus, viruses, zoonoses, avian influenza, influenza

## Abstract

Highly pathogenic avian influenza A(H5N1), clade 2.3.2.1a, with an H9-like polymerase basic protein 1 gene, isolated in Bhutan in 2012, replicated faster in vitro than its H5N1 parental genotype and was transmitted more efficiently in a chicken model. These properties likely help limit/eradicate outbreaks, combined with strict control measures.

In India and Bangladesh, highly pathogenic avian influenza (HPAI) A(H5N1) viruses of the 2.3.2.1a genetic lineage have circulated in poultry since 2011 (*1**–**3*). Subtype H5N1 endemicity is complicated by co-circulating subtype H9N2, G1_Mideast lineage (*4*,*5*), which derives 5 internal genes from HPAI A(H7N3) virus from Pakistan (*4*). A reassortant H5N1 2.3.2.1a virus, rH5N1, with an H9N2-like polymerase basic protein 1 (PB1) gene (H7N3 origin), was reported in Bangladesh (*2**,**5**,**6*), India, and Nepal (*7*). However, its virulence and transmissibility are undetermined.

In Bhutan, the poultry sector consists of free-range backyard chickens, a rising number of commercial chicken farms, and domestic waterfowl in the south (*8**,**9*). Live-bird markets do not exist, but live birds are imported from India (*8**,**9*).

Bhutan’s poultry sector was severely affected by outbreaks of HPAI A(H5N1) clade 2.3.2.1 virus infection during 2012–2013 (*10*). Veterinary authorities enforced strict control measures, including depopulation of poultry in affected regions and burning of related housing and equipment (*11*). Illegal movement of poultry was the major source of outbreaks (*11*). Although the introduction of HPAI A(H5N1) from neighboring H5N1-endemic countries is a constant threat, the subtype is not yet entrenched in poultry in Bhutan.

## The Study

**Figure 1:**
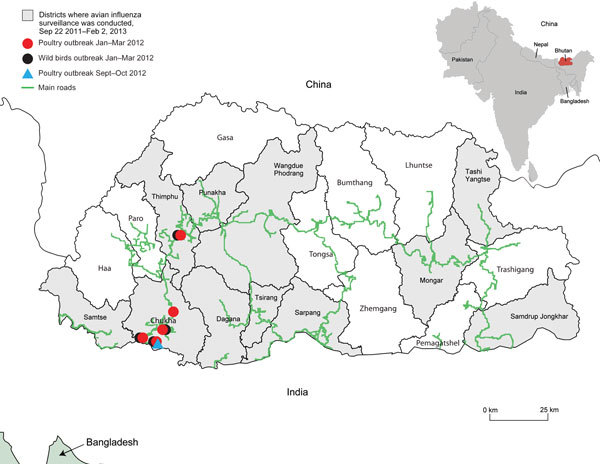
Locations of outbreaks of highly pathogenic avian influenza (H5N1) virus, Bhutan, 2011–2013.

**Figure 2:**
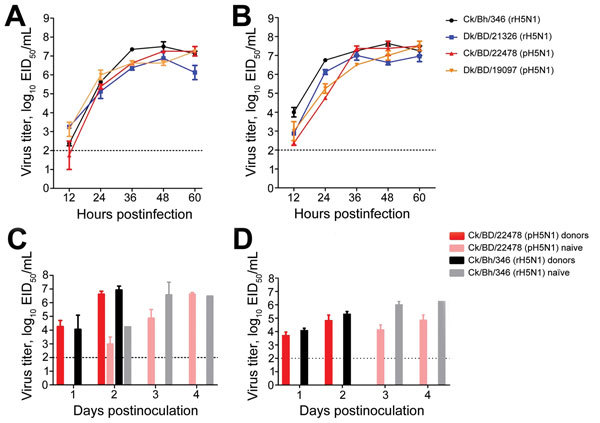
Pathogenesis of influenza virus rH5N1 and pH5N1 2.3.2.1a genotypes in vitro and in vivo. A) Replication kinetics of rH5N1 and pH5N1 in Madin-Darby canine kidney (mammalian) cells. B) Replication kinetics of rH5N1 and pH5N1 in chicken embryonic fibroblast (avian) cells. C) Oropharyngeal shedding and transmissibility of rH5N1 and pH5N1 in a single-virus transmission model in 5-week-old White Leghorn chickens. D) Cloacal shedding and transmissibility of rH5N1 and pH5N1 in a single-virus transmission model in 5-week-old White Leghorn chickens. Naive chickens were co-housed with donors infected with either Ck/22478 (pH5N1) or Ck/Bh/346 (rH5N1) (C and D). The dashed line in each panel represents the limit of virus detection. ANOVA, analysis of variance; Ck/22478, A/chicken/Bangladesh/22478; Ck/Bh/346, A/chicken/Bhutan/346/2012; Dk/BD/23126, A/duck/Bangladesh/23126; Dk/BD/19097/2013, A/duck/Bangladesh/19097; EID, egg infectious dose; dpi, days postinoculation; hpi, hours postinfection; pH5N1, pandemic H1N1; rH5N1, reassortant H5N1.

We isolated HPAI A(H5N1) viruses from samples from 36 chickens and 9 wild birds in Bhutan, all from affected backyard farms adjacent to the highway connecting India with the capital, Thimphu (Figure 1; Technical Appendix 1 Table 1). Phylogenetic analysis (Technical Appendix 1) suggested that the 2012–2013 outbreaks in Bhutan were caused by the rH5N1 genotype (2.3.2.1a/H9-like PB1 [H7N3 origin]), reported in Bangladesh and India at that time (Technical Appendix 1 Figures 1, 2; other data not shown). PB1 phylogeny suggested that this genotype underwent 4 independent reassortment events on the Indian subcontinent (Technical Appendix 1 Figure 2).

Antigenic analysis of selected H5N1 isolates from Bhutan (Technical Appendix 1) showed homogeneity and a reactivity pattern similar to that of H5N1 reference viruses from Bangladesh (Table). Amino acid differences were observed between strains A/chicken/Bhutan/346/2012 (Ck/Bh/346) (rH5N1) and A/chicken/Bangladesh/22478/2014 (Ck/BD/22478), representing the parental H5N1 clade 2.3.2.1a genotype (pH5N1) (Technical Appendix 1 Table 2).

**Table:** Results of hemagglutination inhibition assays of highly pathogenic avian influenza H5N1 viruses isolated in Bhutan, 2012*

Antigens	Genetic clade	Postinfection ferret antisera				
		α-A/BHG/QH/IA clade 2.2	α-A/Hubei/1/2010 clade 2.3.2.1a	α-A/ck/BD/15205 clade 2.3.2.1a	α-A/dk/BD/19097 clade 2.3.2.1a†	α-A/ck/Bhutan/346 clade 2.3.2.1a†
Reference antigens						
A/BHG/QH/IA	2.2	**320**	40	40	80	40
A/Hubei/1/2010	2.3.2.1a	40	**640**	160	1280	80
A/ck/BD/15205	2.3.2.1a	10	80	**80**	320	40
A/dk/BD/19097	2.3.2.1a	-	40	80	**320**	40
A/ck/Bhutan/346	2.3.2.1a	10	40	80	640	**80**
Test antigens						
A/chicken/Bhutan/257/2012	2.3.2.1a	20	40	40	640	40
A/chicken/Bhutan/260/2012	2.3.2.1a	20	40	80	640	80
A/wild bird/Bhutan/357/2012	2.3.2.1a	20	40	80	640	40
A/chicken/Bhutan/1026/2012	2.3.2.1a	40	40	80	1280	80
A/chicken/Bhutan/1030/2012	2.3.2.1a	80	160	320	1280	320
A/chicken/Bhutan/317/2012	2.3.2.1a	10	40	80	640	80
A/wild bird/Bhutan/326/2012	2.3.2.1a	10	80	40	320	20
A/wild bird/Bhutan/328/2012	2.3.2.1a	40	20	40	640	80
A/wild bird/Bhutan/356/2012	2.3.2.1a	40	160	160	640	80
A/chicken/Bhutan/406/2012	2.3.2.1a	20	40	80	320	80
A/chicken/Bhutan/413/2012	2.3.2.1a	20	40	80	640	40
A/chicken/Bhutan/505/2012	2.3.2.1a	80	40	80	640	80
A/chicken/Bhutan/933/2012	2.3.2.1a	40	40	80	640	80
GMT (95% CI)		27.54 (18.36–41.30)	49.51 (34.61–70.83)	80 (56.83–112.6)	640 (502.5–815.1)	68.17 (46.24–100.5)

*Boldface indicates homologous titers. A/BHG/QH/IA, A/bar-headed goose/Qinghai/IA/2005; A/ck/BD/15205, A/chicken/Bangladesh/15205/2012; A/ck/Bhutan/346, A/chicken/Bhutan/346/2012; A/dk/BD/19097, A/duck/Bangladesh/19097/2013; GMT, geometric mean titer. †The immune response in ferrets was boosted with Freund’s incomplete adjuvant (InvivoGen, San Diego, CA, USA) at day 14 postinfection.

To assess whether the rH5N1-PB1 gene conferred a fitness advantage over the pH5N1 genotype, we examined replication kinetics in vitro (Technical Appendix 1). The replication patterns of rH5N1 and pH5N1 were similar in Madin-Darby canine kidney (mammalian) cells (Figure 2, panel A). However, in chicken embryo fibroblasts (CEFs), Ck/Bh/346 (rH5N1) titers were significantly higher than those of Dk/BD/21326 (rH5N1) (p<0.05) and Ck/BD/22478 (pH5N1) (p<0.01) at 12 hours postinoculation (hpi) and those of Ck/BD/22478 (pH5N1) (p<0.001), and Dk/BD/19097 (pH5N1) (p<0.01) at 24 hpi. Dk/BD/21326 (rH5N1) had significantly higher titers than did Ck/BD/22478 (pH5N1) (p<0.01) at 24 hpi (Figure 2, panel B). These results suggest rH5N1 viruses have a selective growth advantage in avian cells at early time points.

Next, we examined whether this growth advantage reflected higher pathogenicity or transmissibility for Ck/Bh/346 (rH5N1) in chickens than did Ck/BD/22478 (pH5N1) (online Technical Appendix 1). The 50% lethal dose (LD_50_) for chicken was 16 EID_50_ (50% egg infective dose) for Ck/Bh/346 (rH5N1) and 50 EID_50_ for Ck/BD/22478 (pH5N1). After inoculation with 30 LD_50_ and cohousing with naive contacts, all donors shed virus oropharyngeally and cloacally (Figure 2, panels C, D). All Ck/Bh/346 (rH5N1) donors died within 48 hpi, whereas only 50% of chickens inoculated with Ck/BD/22478 (pH5N1) died. Naive chickens in contact with donors inoculated with Ck/Bh/346 (rH5N1) or Ck/BD/22478 (pH5N1) became infected by day 2 after contact (Figure 2, panel C), started shedding cloacally on day 3 (Figure 2, panel D), and died by day 4. On day 3 after contact, Ck/Bh/346 (rH5N1) contacts had oropharyngeal and cloacal titers >1 log_10_ EID_50_/mL higher than those of Ck/BD/22478 (pH5N1) contacts (Figure 2, panels C, D), but the difference was not significant.

We placed Ck/Bh/346 (rH5N1) and Ck/BD/22478 (pH5N1) in direct competition by co-housing chickens inoculated with each virus with naive contacts (Technical Appendix 1). All donors shed virus oropharyngeally and cloacally, starting at 1 day postinoculation (dpi). By day 3 after contact, real-time reverse transcription PCR to detect PB1 (Technical Appendix 1) revealed that 7 of 8 naive contacts simultaneously exposed to both viruses were infected with Ck/Bh/346 (rH5N1) alone, none was infected with Ck/BD/22478 (pH5N1) alone, and 1 was co-infected with both viruses. Thus, despite the lower infectious dose used for 30 LD_50_, Ck/Bh/346 (rH5N1) killed inoculated chickens faster than did Ck/BD/22478 (pH5N1) and was transmitted faster and more efficiently to naive contacts.

We assessed the risk for human infection with rH5N1 by investigating its pathogenicity and transmissibility in ferrets (Technical Appendix 1). Donors shed 4.5 log_10_ EID_50_/mL and 3.4 log_10_ EID_50_/mL in nasal wash samples at 2 dpi and 4 dpi, respectively, but cleared the virus by 6 dpi. No direct or aerosol contacts shed virus, suggesting that Ck/Bh/346 (rH5N1) was not transmitted (data not shown). No Ck/Bh/346 (rH5N1)–inoculated ferrets lost >5% of their body weight or showed elevated body temperature (data not shown). Histopathologic analysis showed that 1 donor, who was lethargic at 3-10 dpi, had mild meningoencephalitis at 14 dpi (Technical Appendix 2). A nucleocapsid protein–positive cell was detected in the brain, suggesting that Ck/Bh/346 (rH5N1) is neurotropic. The other ferrets showed no clinical signs of disease. Virus replication was detected in the lung at 4 dpi (log_10_ 2.75 EID_50_/g) (Technical Appendix 2).

## Conclusions

Our study revealed that the viruses that caused the 2012 outbreaks in Bhutan belonged to the rH5N1 genotype (2.3.2.1a/H9-like PB1 [7:1]), whereas neither H9N2 nor the pH5N1 genotype have been detected there. rH5N1 has been isolated sporadically at live-bird markets and from chickens on farms where outbreaks occurred in Bangladesh (*5*,*6*), India (*12*), and Nepal (*7*) in 2011–2013. The lack of data on the effect of the H9-like PB1 gene on the virulence of rH5N1 makes determining its pathogenicity and transmissibility a critical public-health goal for Bhutan and neighboring countries.

Ck/Bh/346 (rH5N1) killed inoculated chickens faster than did Ck/BD/22478 (pH5N1), despite the lower infectious dose used for Ck/Bh/346. In CEFs, Ck/Bh/346replicated with greater efficiency during the first 36 hpi than did Ck/BD/22478, which possibly explains why rH5N1 transmits more efficiently to naive chickens when directly competing with pH5N1. How faster replication contributes to the increased mortality rate of naive chickens might be crucial to eradicating the disease in Bhutan. In a mountainous region with widely separated villages, small-scale poultry farming, and no live-bird markets, the severity and rapid onset of the infection could lead to host-resource exhaustion and self-limitation.

To determine whether the reassortant PB1 gene accounts for the observed phenotypic properties of rH5N1, reverse genetics experiments are required. Despite its enhanced transmissibility, rH5N1 did not supplant pH5N1 in India or Bangladesh after undergoing multiple reassortment events. Possible reasons for this include the involvement of other influenza subtypes, which would complicate the competition/transmission model, especially at live-bird markets, as well as the large duck population, which is prone to inapparent HPAI infection (indicating possible underreporting).

Our ferret model results suggest that avian-to-human transmission of rH5N1 is possible. However, further adaptation to the host is necessary for rH5N1 to become transmissible among mammals. Similar results have been reported for H5N1 clade 2.3.2.1 (*13*), H5N1 clade 2.3.4 (*14*), and H5Nx clade 2.3.4.4 (*15*). rH5N1 is potentially neurotropic, manifesting clinically as mild meningoencephalitis with no obvious respiratory involvement. This finding has implications on early diagnosis and the use of antiviral drugs during the first 48 h of clinical onset for optimal therapeutic effect.

pH5N1 and H9N2 virus strains will likely continue to co-circulate on the Indian subcontinent, enabling further reassortment events. Our results highlight the need for active surveillance and full-genome sequencing of all H5N1 virus isolates.

Technical Appendix 1 Methods of isolating highly pathogenic avian influenza A(H5N1) viruses from chickens and wild birds in Bhutan, 2012–2013, and details of genotypic analysis.

Technical Appendix 2 Results of histopathologic examination and immunohistochemical analysis of ferret tissues after infection with influenza A/chicken/Bhutan/346/2012 (reassortant H5N1).
