# Supplementary material for: Highly Pathogenic Reassortant Avian Influenza A(H5N1) Virus Clade 2.3.2.1a in Poultry, Bhutan
Source: Emerg Infect Dis. 2016 Dec;22(12):2137–41. doi: 10.3201/eid2212.160611 (PMC5189144; doi:10.3201/eid2212.160611)
Supplement: Technical Appendix 1 — Methods of isolating highly pathogenic avian influenza A(H5N1) viruses from chickens and wild birds in Bhutan, 2012–2013, and details of genotypic analysis. [file 16-0611-Techapp-s1.pdf]

Article DOI: http://dx.doi.org/10.3201/eid2212.160611

Highly Pathogenic Reassortant Avian Influenza A(H5N1) Virus Clade 2.3.2.1a in Poultry, Bhutan

Technical Appendix 1

All experiments were conducted in enhanced animal biosafety level 3 (ABSL3+) facilities at St. Jude Children's Research Hospital (St. Jude, Memphis, TN, USA) and were approved by the institutional Animal Care and Use Committee.

Sample Collection and Virus Isolation

From September 22, 2011, to February 2, 2013, oropharyngeal and cloacal swab samples from poultry (1,005 chickens and 41 ducks) and 698 fecal samples from wild birds (including the black-necked crane [*Grus nigricollis*], the cattle egret [*Bubulcus ibis*], the yellow-billed duck [*Anas undulata*], and other terrestrial wild birds) were collected from 11 districts in Bhutan (Figure 1). Samples were stored in 1 mL of glycerol medium (*1*), transported to St. Jude, and injected into 10-day-old embryonated chicken eggs for virus isolation (*2*). The presence of influenza A virus (IAV) in allantoic fluids testing positive for hemagglutination was confirmed by endpoint real-time reverse transcription PCR (RT-PCR) (to detect the M gene) and sequencing (*3*).

Serologic Testing

Fourteen representative subtype H5N1 viruses from Bhutan were selected for use in hemagglutination-inhibition (HI) tests (*4*), on the basis of their genetic relationships (nucleotide differences in the hemagglutinin (HA) gene), as well as the location and date of their isolation. Postinfection ferret antisera from A/H5N1 clades 2.2 and 2.3.2.1, including recent isolates from Bhutan and Bangladesh (Table), were produced as described (*5*). Horse red blood cells (1% + 0.5% bovine serum albumin; Sigma, Saint Louis, MO, USA) were used for HI tests (*6*).

Viral Genome Sequencing and Phylogenetic Analysis

Full-genome sequencing was performed at the Hartwell Center for Bioinformatics and Biotechnology at St. Jude, as described (*5*). Gene sequences obtained were submitted to GenBank under accession nos. KJ682226–KJ682315 and KX215199–KX215468. The Lasergene package (DNASTAR, Madison, WI, USA) was used for sequence analysis. Phylogenetic analyses of full-length nucleotide sequences were conducted by the maximum-likelihood method, using the Tamura-Nei nucleotide substitution model with 1,000 bootstrap replicates in the MEGA 5 software (*7*). Reference sequences of all available rH5N1 viruses from the Indian subcontinent (as of February 1, 2016) were retrieved from GenBank (*8*) and GISAID EpiFlu (*9*).

Virus Selection

A/chicken/Bhutan/346/2012 (Ck/Bh/346) was selected as the representative virus, because all rH5N1 isolates were genetically and antigenically homogeneous. For in vivo experiments in chickens, Ck/Bh/346(rH5N1) was paired with A/chicken/Bangladesh/22478/2014 (Ck/BD/22478), which represents the pH5N1 genotype that is circulating widely in the Indian subcontinent. For in vitro replication experiments, 2 viruses of duck origin, rH5N1 A/duck/Bangladesh/21326/2013 (Dk/BD/21326) and pH5N1 A/duck/Bangladesh/19097/2013 (Dk/BD/19097), were added.

Growth Kinetics

Virus replication kinetics was determined in MDCK cells and UMNSAH/DF-1 chicken embryo fibroblasts (CEFs) (ATCC, Manassas, VA, USA). Briefly, MDCK cells and CEFs grown in duplicate wells of 6-well plates were inoculated with Ck/Bh/346(rH5N1), Ck/BD/22478(pH5N1), Dk/BD/21326(rH5N1), or Dk/BD/19097(pH5N1) (at a multiplicity of infection of 0.001 PFU/cell). The supernatant was harvested 12–60 hours postinoculation (hpi) and titrated in embryonated chicken eggs with 50% egg infective doses (EID_{50}). Virus titers were calculated by the Reed and Muench method (*10*).

Pathogenicity and Transmissibility in Chickens

Five-week-old specific-pathogen-free White Leghorn chickens (3 chickens/group; 6 groups/virus) were used to determine the 50% lethal dose (LD_{50}) of Ck/Bh/346(rH5N1) and Ck/BD/22478(pH5N1) by the Reed and Muench method (*10*). LD_{50} titers were expressed as the EID_{50} value corresponding to 1 LD_{50}.

Chickens (n = 4) were inoculated with 30 LD_{50}/0.5 mL of Ck/Bh/346(rH5N1) or Ck/BD/22478(pH5N1) by the oral, intraocular, or intranasal route, and at 1 hpi, donor birds were cohoused with naïve contacts (n = 2). Surviving donors were removed from the cage at 48 hpi to avoid further exposure to contact birds. Oropharyngeal and cloacal swabs were collected daily for 4 days and titrated in eggs as described above. Morbidity and mortality were monitored twice daily. Competitive virus transmission was examined in a similar setup, except that 2 donor birds were inoculated with Ck/Bh/346(rH5N1) and the other 2 with Ck/BD/22478(pH5N1). The experiment was conducted in 4 replicates in separate isolators. All chickens were screened daily for the presence of virus.

Genotype Determination by Real-Time RT-PCR

Genotypic analysis of competitive virus transmission was performed by multiplex real-time RT-PCR by using viral RNA extracted from individual swabs. Briefly, a set of primers was designed to match the PB1 gene sequence of both the pH5N1 and rH5N1 genotypes (PB1–1462F 5′-GAAGTCTTACATAAATCGGACAGG-3′ and PB1 1640R 5′-GTCCTTGATGAATAGCTGAAGAGC-3′). One probe was designed to match the PB1 sequence of pH5N1 (PB1–1544R 5′-/5HEX/CCACTCCAA/ZEN/AACTGGGCAGCTCCATACTG/3IABkFQ/-3′) and another to match the rH5N1 PB1 sequence (PB1 1568R 5′-/56-FAM/CTCATGTCA/ZEN/GCTGACTCGTTAATCCCAGAT/3IABkFQ/-3′). Cycling conditions were as previously described (*11*). The cutoff cycle threshold (CT) value for rRT-PCR was 40. The specificity of the 2 probes for the viruses used in the study [Ck/Bh/346 (rH5N1), Ck/BD/22478 (pH5N1), Dk/BD/21326 (rH5N1), and Dk/BD/19097 (pH5N1)] was tested. Each probe was able to detect only the RNA of the 2 virus strains that belong to the respective genotype for which it was designed.

Pathogenicity and Transmissibility in Ferrets

Nine 3- to 5-month-old male ferrets (Marshall Farms) that were seronegative by the HI test for currently circulating influenza A/H1N1, H3N2, H5N1, and influenza B viruses were used in the transmission model as previously described (*12*). Briefly, 3 donor ferrets were inoculated intranasally with 10^6 EID_{50}/0.5 mL of Ck/Bh/346(rH5N1), then each of them was cohoused with a separate naïve direct-contact and a naïve aerosol-contact ferret. Control ferrets were mock-

inoculated with sterile phosphate-buffered saline (PBS). Nasal wash specimens from all ferrets were collected every other day after inoculation, and the virus was titrated in eggs. Clinical signs of infection, body temperature, and weight were recorded daily for 14 days.

One ferret inoculated with Ck/Bh/346 was euthanized at 4, 6, and 14 days postinoculation (dpi). Nasal turbinates, trachea, lung, heart, thymus, spleen, small and large intestines, brain, cerebellum, and spinal cord were collected and homogenized in PBS with antibiotics. The virus titer (EID_{50}/g tissue) was then determined in eggs. Histopathologic and immunohistochemical analyses to detect the IAV nucleoprotein were performed as described (*12,13*).

Statistical Analysis

Statistical significance ($p \leq 0.05$) between groups was determined by 2-way analysis of variance (ANOVA), performed by using the Prism 5 software (GraphPad, La Jolla, CA, USA).

References

1. World Health Organization. Manual on animal influenza diagnosis and surveillance. Geneva: WHO Global Influenza Programme; 2002. p. 16–17.

2. Shortridge KF, Butterfield WK, Webster RG, Campbell CH. Isolation and characterization of influenza A viruses from avian species in Hong Kong. Bull World Health Organ. 1977;55:15–20. PubMed

3. Negovetich NJ, Feeroz MM, Jones-Engel L, Walker D, Alam SMR, Hasan MK, et al. Live bird markets of Bangladesh: H9N2 viruses and the near absence of highly pathogenic H5N1 influenza. PLoS ONE. 2011;6:e19311. PubMed http://dx.doi.org/10.1371/journal.pone.0019311

4. Palmer DFDW, Coleman MT, Schild GC. Advanced laboratory techniques for influenza diagnosis. Washington, DC: US Department of Health, Education and Welfare; 1975.

5. Marinova-Petkova A, Feeroz MM, Alam SMR, Hasan MK, Akhtar S, Jones-Engel L, et al. Multiple introductions of highly pathogenic avian influenza H5N1 viruses into Bangladesh. Emerg Microbes Infect. 2014;3:e11.

6. World Health Organization. WHO manual on animal influenza diagnosis and surveillance. Geneva: the Organization; 2011. http://whqlibdoc.who.int/publications/2011/9789241548090_eng.pdf

7. Tamura K, Peterson D, Peterson N, Stecher G, Nei M, Kumar S. MEGA5: molecular evolutionary genetics analysis using maximum likelihood, evolutionary distance, and maximum parsimony methods. Mol Biol Evol. 2011;28:2731–9. PubMed http://dx.doi.org/10.1093/molbev/msr121

8. Benson DA, Karsch-Mizrachi I, Lipman DJ, Ostell J, Wheeler DL. GenBank. Nucleic Acids Res. 2005;33:D34–8. PubMed http://dx.doi.org/10.1093/nar/gki063

9. Bogner P, Capua I, Lipman DJ, Cox NJ, et al. A global initiative on sharing avian flu data. Nature. 2006;442:981. http://dx.doi.org/10.1038/442981a

10. Reed LJ, Muench H. A simple method for estimating fifty percent endpoints. Am J Hyg. 1938;27:493–7.

11. World Health Organization. CDC protocol of real-time RT-PCR for influenza A (H1N1). Geneva: the Organization; 2009. http://www.who.int/csr/resources/publications/swineflu/CDCRealtimeRTPCR_SwineH1Assay-2009_20090430.pdf?ua=1

12. Yen HL, Liang CH, Wu CY, Forrest HL, Ferguson A, Choy KT, et al. Hemagglutinin-neuraminidase balance confers respiratory-droplet transmissibility of the pandemic H1N1 influenza virus in ferrets. Proc Natl Acad Sci U S A. 2011;108:14264–9. PubMed http://dx.doi.org/10.1073/pnas.1111000108

13. Govorkova EA, Rehg JE, Krauss S, Yen HL, Guan Y, Peiris M, et al. Lethality to ferrets of H5N1 influenza viruses isolated from humans and poultry in 2004. J Virol. 2005;79:2191–8. PubMed http://dx.doi.org/10.1128/JVI.79.4.2191-2198.2005

Technical Appendix 1 Table 1. Field data for highly pathogenic reassortant avian influenza A/H5N1 (rH5N1)–positive samples collected in Bhutan in 2012

Isolate no.	Collection date	Location state	Location	Habitat	Isolate name
1	01/08/2012	Thimphu	Thimphu	Farm, free-range	A/chicken/Bhutan/367/2012
2	01/15/2012	Chukha	Bjabchu	Farm, free-range	A/chicken/Bhutan/317/2012
3	01/16/2012	Thimphu	Changgidaphu	Farm, free-range	A/chicken/Bhutan/406/2012
4	01/16/2012	Thimphu	Changgidaphu	Farm, free-range	A/chicken/Bhutan/407/2012
5	01/23/2012	Chukha	N/A	Farm, free-range	A/chicken/Bhutan/505/2012
6	01/23/2012	Chukha	N/A	Farm, free-range	A/chicken/Bhutan/507/2012
7	01/27/2012	Chukha	Phuntsholing	Unknown	A/wild bird/Bhutan/325/2012
8	01/27/2012	Chukha	Phuntsholing	Unknown	A/wild bird/Bhutan/326/2012
9	01/27/2012	Chukha	Phuntsholing	Unknown	A/wild bird/Bhutan/327/2012
10	01/27/2012	Chukha	Phuntsholing	Unknown	A/wild bird/Bhutan/328/2012
11	01/27/2012	Chukha	Phuntsholing	Unknown	A/wild bird/Bhutan/329/2012
12	01/27/2012	Chukha	Phuntsholing	Farm, free-range	A/chicken/Bhutan/330/2012
13	01/27/2012	Chukha	Phuntsholing	Farm, free-range	A/chicken/Bhutan/331/2012
14	01/29/2012	Thimphu	Thimphu	Farm, free-range	A/chicken/Bhutan/345/2012
15	01/29/2012	Thimphu	Thimphu	Farm, free-range	A/chicken/Bhutan/346/2012
16	01/29/2012	Thimphu	Thimphu	Farm, free-range	A/chicken/Bhutan/347/2012
17	01/29/2012	Thimphu	Thimphu	Farm, free-range	A/chicken/Bhutan/349/2012
18	01/30/2012	Chukha	Wangdigatsel	Farm, free-range	A/chicken/Bhutan/352/2012
19	01/30/2012	Chukha	Wangdigatsel	Farm, free-range	A/wild bird/Bhutan/356/2012
20	01/30/2012	Chukha	Bjabchu	Farm, free-range	A/yellow billed duck/Bhutan/358/2012
21	01/30/2012	Chukha	Bjabchu	Farm, free-range	A/chicken/Bhutan/359/2012
22	01/31/2012	Thimphu	Thimphu	Unknown	A/wild bird/Bhutan/357/2012
23	02/11/2012	Thimphu	Thimphu	Farm, free-range	A/chicken/Bhutan/297/2012
24	02/11/2012	Thimphu	Thimphu	Farm, free-range	A/chicken/Bhutan/298/2012
25	02/14/2012	Thimphu	Thimphu	Farm, free-range	A/chicken/Bhutan/257/2012
26	02/14/2012	Thimphu	Thimphu	Farm, free-range	A/chicken/Bhutan/258/2012
27	02/14/2012	Thimphu	Thimphu	Farm, free-range	A/chicken/Bhutan/259/2012
28	02/14/2012	Thimphu	Thimphu	Farm, free-range	A/chicken/Bhutan/260/2012
29	02/14/2012	Chukha	Phuntsholing, Balujhora	Farm, free-range	A/chicken/Bhutan/265/2012
30	02/18/2012	Chukha	Gedu	Farm, free-range	A/chicken/Bhutan/413/2012
31	02/18/2012	Chukha	Gedu	Farm, free-range	A/chicken/Bhutan/414/2012
32	02/18/2012	Chukha	Gedu	Farm, free-range	A/chicken/Bhutan/415/2012
33	02/18/2012	Chukha	Gedu	Farm, free-range	A/chicken/Bhutan/416/2012
34	02/18/2012	Chukha	Gedu	Farm, free-range	A/chicken/Bhutan/417/2012
35	02/18/2012	Chukha	Gedu	Farm, free-range	A/chicken/Bhutan/418/2012
36	02/29/2012	Chukha	Gedu	Unknown	A/wild bird/Bhutan/308/2012
37	03/18/2012	Thimphu	Thimphu	Farm, free-range	A/chicken/Bhutan/1026/2012
38	03/18/2012	Thimphu	Thimphu	Farm, free-range	A/chicken/Bhutan/1028/2012
39	03/18/2012	Thimphu	Thimphu	Farm, free-range	A/chicken/Bhutan/1029/2012
40	03/18/2012	Thimphu	Thimphu	Farm, free-range	A/chicken/Bhutan/1030/2012
41	03/18/2012	Thimphu	Thimphu	Farm, free-range	A/chicken/Bhutan/1031/2012
42	09/10/2012	Chukha	Phuntsholing, Rinchending	Unknown	A/chicken/Bhutan/933/2012
43	09/10/2012	Chukha	Phuntsholing, Rinchending	Unknown	A/chicken/Bhutan/934/2012
44	10/11/2012	Chukha	Phuntsholing, Rinchending	Unknown	A/chicken/Bhutan/935/2012
45	10/11/2012	Chukha	Phuntsholing, Rinchending	Unknown	A/chicken/Bhutan/936/2012

Technical Appendix 1 Table 2. Amino acid differences in the genomes of A/chicken/Bangladesh/22478/2014 (pH5N1) and A/chicken/Bhutan/346/2012 (rH5N1)

		Amino acid positions																									
Gene	Virus	64	299	339	344	345	355	457	553	702																	
PB2	Ck/BD/22478	I	K	I	V	F	R	I	V	K																	
	Ck/Bh/346	M	R	T	L	L	K	V	I	R																	
		11	12	14*	20	54	105	149	157	158†	191	215 †	261	384	386	398	423	429	456	515	578	584	621	635	644	667	
PB1	Ck/BD/22478	R	V	V	T	E	T	I	A	N	V	R	S	I	K	D	I	K	R	A	R	H	Q	R	I	T	
	Ck/Bh/346	K	I	A	I	R	N	V	V	S	I	K	T	T	R	N	V	R	H	S	K	R	R	K	V	I	
		6	7	13	16	18	20	21	22	23	27	30	33	35	36	37	40	48	50	57	60	70	74	75	77	79	84
PB1-F2	Ck/BD/22478	G	I	T	T	I	K	K	V	S	T	Q	P	S	T	R	D	P	G	Y	Q	E	L	R	L	R	N
	Ck/Bh/346	D	T	I	I	T	R	R	G	D	I	L	L	L	I	Q	G	Q	D	C	R	G	T	H	S	L	S
		20	57	59	61	118	231	405	425	614																	
PA	Ck/BD/22478	A	R	D	T	V	T	C	F	A																	
	Ck/Bh/346	T	G	E	I	I	A	S	L	T																	
		95	121	156	174	189	236	266	320	396	523																
HA‡§	Ck/BD/22478	L	F	T	I	R	K	R	N	V	A																
	Ck/Bh/346	F	S	A	V	M	N	K	S	I	V																
		8	13	19	35	100	346	373	382	434																	
NA¶	Ck/BD/22478	I	V	T	G	H	V	I	D	G																	
	Ck/Bh/346	M	I	I	S	Y	I	M	G	S																	
		82																									
M2	Ck/BD/22478	N																									
	Ck/Bh/346	S																									
		76	114																								
NS1#	Ck/BD/22478	G	P																								
	Ck/Bh/346	E	S																								
		26	48																								
NS2	Ck/BD/22478	G	M																								
	Ck/Bh/346	D	V																								

*Amino acid substitution V14A in the PB1 has been associated with reduced polymerase activity and reduced transmissibility in chickens in recombinant influenza A viruses with HA and NA from highly pathogenic H5N1 and internal genes from low pathogenicity viruses (Suzuki Y, Uchida Y, Tanikawa T, Maeda N, Takemae N, Saito T. Amino acid substitutions in PB1 of avian influenza viruses influence pathogenicity and transmissibility in chickens. J Virol. 2014;88:11130–9).
†N158S and R215K have been reported to potentially decrease the replication and pathogenicity of reassortant H5N1 viruses [Wasilenko JL, Lee CW, Sarmento L, Spackman E, Kapczynski DR, Suarez DL, et al. NP, PB1, and PB2 viral genes contribute to altered replication of H5N1 avian influenza viruses in chickens. J Virol. 2008;82(9):4544-53].
‡Mature H5 HA.
§The 2 viruses have identical polybasic cleavage-site motifs in the hemagglutinin (PQRERRRK_RGLF).
¶Both viruses have a 20-aa deletion at positions 49–68 of the neuraminidase.
#Both viruses have a 5-aa deletion at positions 80–84 of the NS1 gene.

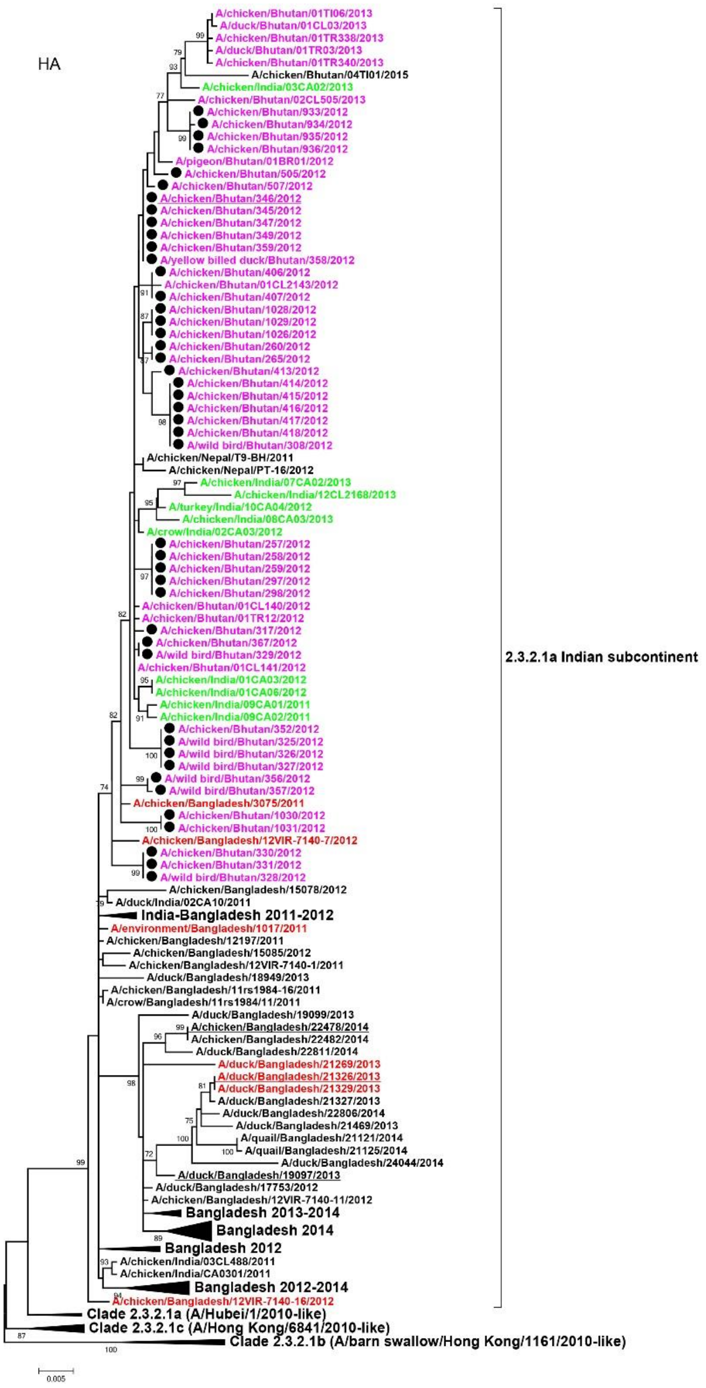

Technical Appendix Figure 1. Phylogenetic relationships of the hemagglutinin gene of highly pathogenic avian influenza (HPAI) (H5N1) viruses isolated in Bhutan. Phylogeny was reconstructed by using the maximum likelihood method based on the Tamura-Nei model and with 1,000 bootstrap replicates. Numbers at the branches indicate bootstrap values; only values >70 are shown. HPAI H5N1 viruses from the reassortant genotype are color coded. Red, rH5N1 viruses isolated in Bangladesh; green, rH5N1 viruses isolated in India; pink, rH5N1 viruses isolated in Bhutan; black circle, viruses isolated during this study. Virus strains that are underlined were used for in vitro or in vivo experiments.

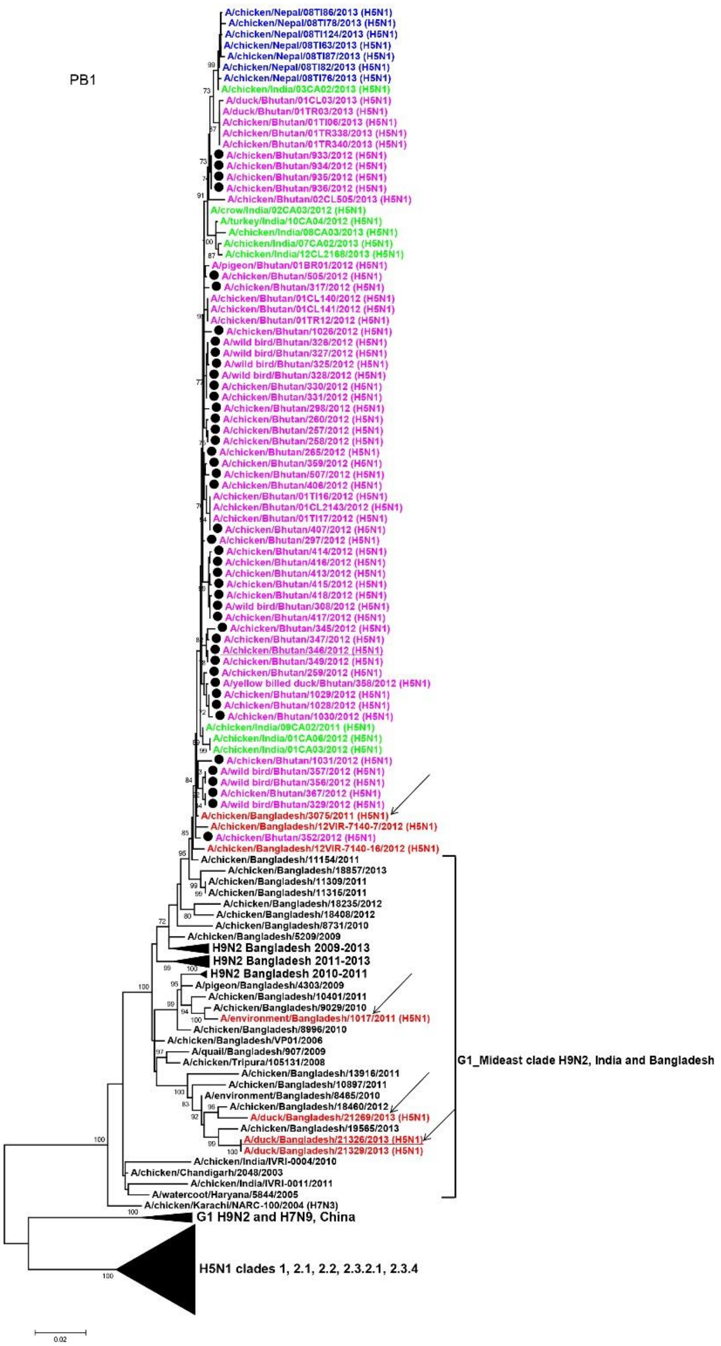

Technical Appendix Figure 2. Phylogenetic relationships of the PB1 gene of highly pathogenic influenza (HPAI) (H5N1) viruses isolated in Bhutan. Phylogeny was reconstructed by using the maximum likelihood method based on the Tamura-Nei model with 1,000 bootstrap replicates. Numbers at the branches indicate bootstrap values; only values >70 are shown. HPAI H5N viruses from the reassortant genotype are color coded. Red, rH5N1 viruses isolated in Bangladesh; green, rH5N1 viruses isolated in India; blue, rH5N1 viruses isolated in Nepal; pink, rH5N1viruses isolated in Bhutan. Black circle shows viruses isolated during this study. Virus strains that are underlined were used for in vitro or in vivo experiments. Arrows show separate reassortment events.
