# Supplementary material for: Highly Pathogenic Reassortant Avian Influenza A(H5N1) Virus Clade 2.3.2.1a in Poultry, Bhutan
Source: Emerg Infect Dis. 2016 Dec;22(12):2137–41. doi: 10.3201/eid2212.160611 (PMC5189144; doi:10.3201/eid2212.160611)
Supplement: Technical Appendix 2 — Results of histopathologic examination and immunohistochemical analysis of ferret tissues after infection with influenza A/chicken/Bhutan/346/2012 (reassortant H5N1). [file 16-0611-Techapp-s2.pdf]

Article DOI: http://dx.doi.org/10.3201/eid2212.160611

Highly Pathogenic Reassortant Avian Influenza A(H5N1) Virus Clade 2.3.2.1a in Poultry, Bhutan

Technical Appendix 2

Results of Histopathologic and Immunohistochemical Analyses

Histopathologic examination of ferrets inoculated intranasally with 10^6 EID_{50}/0.5 mL of Ck/Bh/346(rH5N1) revealed upper and lower respiratory tract involvement (Technical Appendix 2 Figure, center column). Inflammation, degeneration, edema, and necrosis of the respiratory and olfactory epithelium of the nasal cavity, along with generalized pneumonia involving bronchi, bronchioles, and alveoli, occurred at 4 and 6 dpi; however, at 14 dpi the number of lesions was substantially lower. A donor ferret that displayed lethargy between 3 and 10 dpi was euthanized at 14 dpi for tissue collection and was diagnosed post mortem with mild meningoencephalitis with multifocal lesions in the olfactory bulb (Technical Appendix 2 Figure). There were no pathologic changes in other tissues of this animal.

Immunohistochemical analysis showed multiple foci of influenza A virus nucleocapsid protein (NP)–positive epithelial cells in the nasal cavity and the alveolar tissue of the lungs of inoculated ferrets at 4 dpi (Figure, right column), but only scattered individual positive cells at 6 dpi. A single NP-positive cell was detected at 14 dpi in the brain of the ferret with meningoencephalitis (Figure, bottom right).

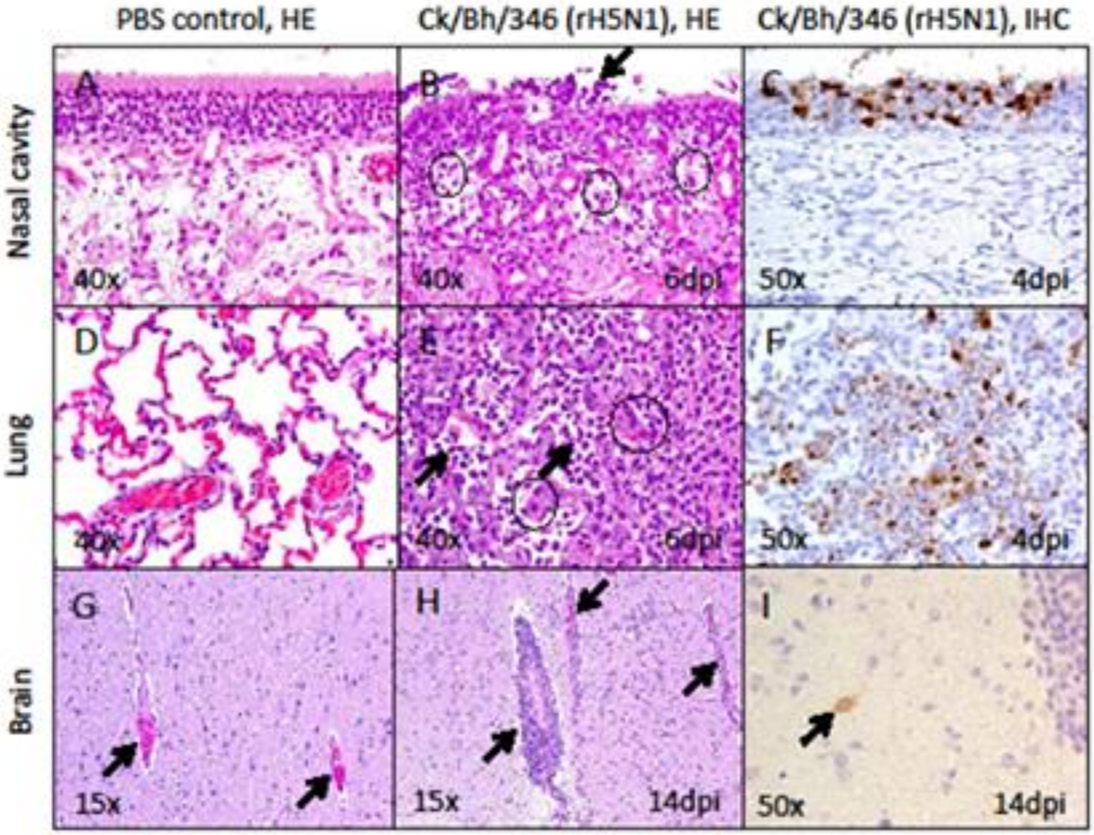

Technical Appendix Figure. Results of histopathologic examination and immunohistochemical analysis of ferret tissues after infection with A/chicken/Bhutan/346/2012 (rH5N1). The images in the left column are tissue sections from a control ferret inoculated with PBS; the images in the middle and right columns are tissue sections from a ferret infected with A/chicken/Bhutan/346/2012 (rH5N1). Magnifications are shown in the bottom left corner of each panel; times postinfection are indicated in the bottom right corner of panels showing infected ferret tissues. A) Normal septal olfactory epithelium and submucosal glands in the nasal cavity of an infected ferret. B) Septal olfactory epithelial necrosis, sloughing (arrow), submucosal inflammation (inflammatory cells are circled), and edema in the nasal cavity of an infected ferret. C) Immunohistochemical analysis of septal olfactory epithelium of an infected ferret. Cells containing the influenza A NP are stained brown. D) Normal alveolar tissue with thin alveolar walls and blood vessels not surrounded by inflammatory cells. E) Alveolitis (inflammation of the alveolar tissue) in an infected ferret, showing septal thickening, hyperplasia, hypertrophy of the pneumocytes (circled), and the presence of large numbers of inflammatory cells (arrows). F) Immunohistochemical analysis of an area of alveolitis in the lung of an infected ferret. Cells containing the influenza A NP are stained brown. G) Normal brain tissue with blood vessels (arrows). H) Encephalitis (inflammation of the brain) in an infected ferret, showing perivascular cuffing (arrows) at the level of the optic chiasma. Note the presence of large numbers of mononuclear inflammatory cells and neutrophils. I) The arrow shows a single influenza A–infected cell (the viral NP is stained brown) in an area of encephalitis in the brain of an

infected ferret. Perivascular cuffing is visible in the upper right corner of the image. rH5N1, reassortant H5N1; HE, hematoxylin-and-eosin staining; NP, nucleocapsid protein; IHC, immunohistochemical staining.
